# Supplementary figures and images for: Comparison of LncRNA Expression Profiles during Myogenic Differentiation and Adipogenic Transdifferentiation of Myoblasts
Source: Int J Mol Sci. 2019 Jul 30;20(15):3725. doi: 10.3390/ijms20153725 (PMC6695777; doi:10.3390/ijms20153725)

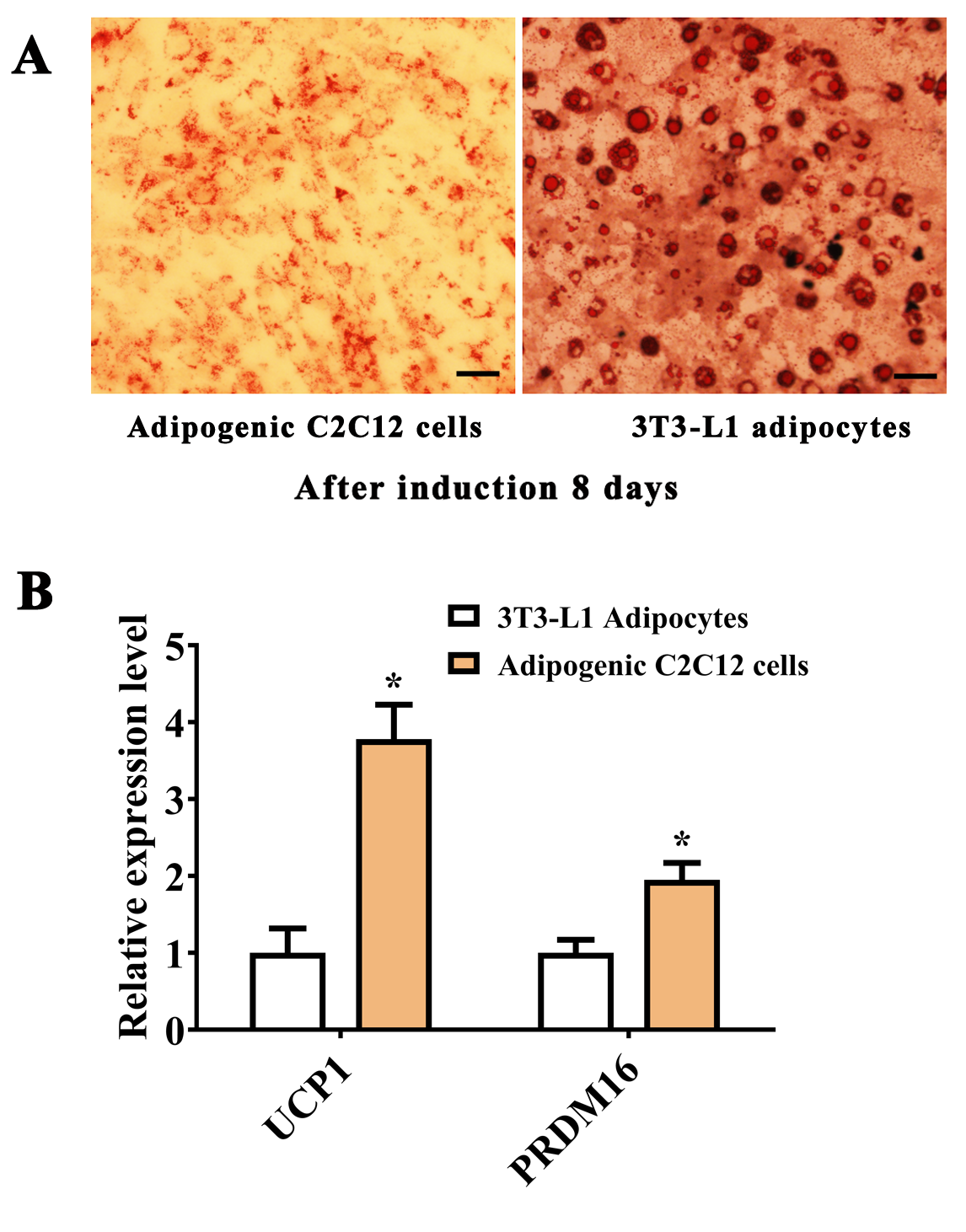

Supplement: Supplementary file 1 [file ijms-20-03725-s001.zip › ijms-547283-SI/Supplementary Files/supplementary Fig 1.tif]

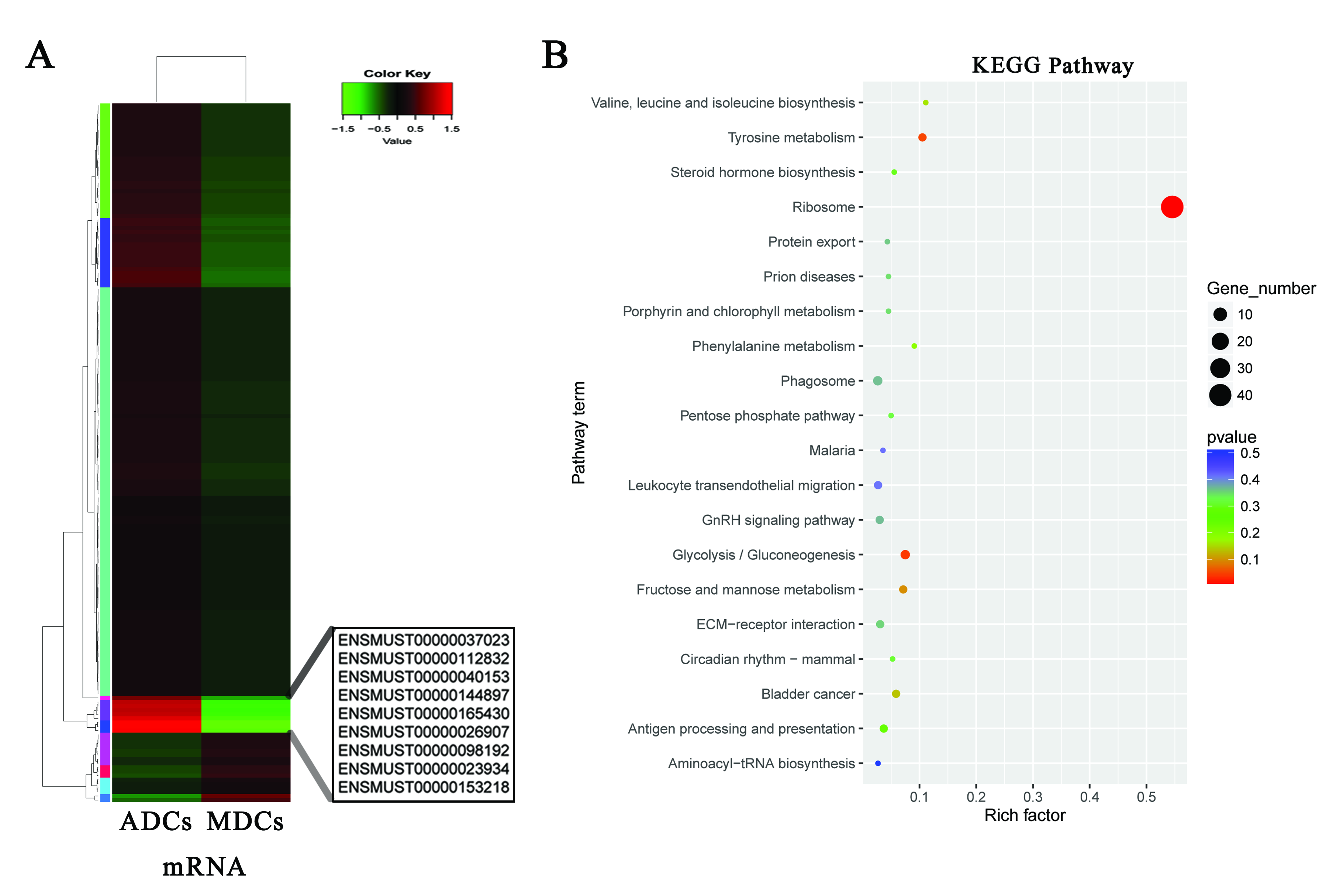

Supplement: Supplementary file 1 [file ijms-20-03725-s001.zip › ijms-547283-SI/Supplementary Files/Supplementary Fig 2.tif]
